# Q-learning model of insight problem solving and the effects of learning traits on creativity

**DOI:** 10.3389/fpsyg.2023.1287624

**Published:** 2024-01-08

**Authors:** Tsutomu Harada

**Affiliations:** Graduate School of Business Administration, Kobe University, Kobe, Japan

**Keywords:** insight problem solving, creativity, individual differences, Q-learning, learning transfer

## Abstract

Despite the fact that insight is a crucial component of creative thought, the means by which it is cultivated remain unknown. The effects of learning traits on insight, specifically, has not been the subject of investigation in pertinent research. This study quantitatively examines the effects of individual differences in learning traits estimated using a Q-learning model within the reinforcement learning framework and evaluates their effects on insight problem solving in two tasks, the 8-coin and 9-dot problems, which fall under the umbrella term “spatial insight problems.” Although the learning characteristics of the two problems were different, the results showed that there was a transfer of learning between them. In particular, performance on the insight tasks improved with increasing experience. Moreover, loss-taking, as opposed to loss aversion, had a significant effect on performance in both tasks, depending on the amount of experience one had. It is hypothesized that loss acceptance facilitates analogical transfer between the two tasks and improves performance. In addition, this is one of the few studies that attempted to analyze insight problems using a computational approach. This approach allows the identification of the underlying learning parameters for insight problem solving.

## Introduction

Creativity occasionally depends on insight, the ability of an individual to alter their existing thought patterns, break the status quo, and create something new without being aware of the process by which the solution was reached. While analytical problems are solved through a step-by-step, incremental process, insight problems require an “a-ha” moment that leads to a solution. The information gained in this way transcends current informational boundaries and contributes to solving the problem.

The underlying mechanisms of creative thinking in which insight plays a critical role, have been the subject of intensive research efforts that have led to a number of studies using a variety of insight tasks as summarized in Table 1. Several conceptual models have been developed as a result, such as the representational change theory (Ohlsson, 1992; Knöblich et al., 1999), the breakthrough thinking model (Perkins, 2000), and “Geneplore” model (Finke et al., 1999). These cognitive models of insight generation appear less reliant on analytical processes. According to these models, attempts to solve problems failed, impasses were reached, restructuring occurred, and the “a-ha” moment led to a solution (Weisberg, 2015). However, some studies have suggested that creativity is identical to analytical problem solving, and that insight and impasse have no influence on it (Weisberg, 2006, 2013; Ball and Stevens, 2009; Chein and Weisberg, 2014). According to this view, insight tasks differ due to their high domain specificity. Therefore, an integrated model has been proposed that includes solutions by transfer, heuristic methods, restructuring, and insight, primarily based on analytic thinking processes (Weisberg, 2015). Although problem solving through insight is the final step, most problems can be solved before reaching an impasse and gaining insight. However, because this integrated model is a categorical stage model, it is difficult to quantitatively assess the relative contribution of analytical thinking and other learning traits to problem solving (for a systematic review of insight problem solving, see van Steenburgh et al., 2012).

**Table 1:** Insight tasks in problem-solving.

Type	Insight tasks	Representative studies
Verbal	(Compound) Remote Associates Test	Mednick and Andrews (1967), Beeman and Bowden (2000), Bowden and Jung-Beeman (2003), and Kounios et al. (2006)
Verbal	Anagram tasks	Kounios et al. (2008), Aziz-Zadeh et al. (2009), and Laukkonen et al. (2020)
Conceptual	Riddles, brainteasers	Luo and Niki (2003) and Qiu et al. (2006)
Conceptual and Spatial	Magic tricks	Danek et al. (2014) and Hedne et al. (2016)
Spatial	9-dot problem	Maier (1930) and Mac Gregor et al. (2001)
Spatial	8-coin problem	Ormerod et al. (2002)
Spatial	Visual tasks	Kizilirmak et al. (2016) and Laukkonen and Tangen (2017)
Spatial	T puzzles	Suzuki et al. (2014)
Spatial & Numerical	Matchsticks arithmetic problems	Knöblich et al. (1999)
Numerical	Number reduction task	Haider and Rose (2007)

This study investigated the contribution of learning traits such as exploitation/exploration trade-offs, risk attitude, and loss aversion to problem solving in insight tasks. Although insight problems can be solved analytically without insight, which is extremely rare under laboratory conditions (Fleck and Weisberg, 2013), solving insight problems requires removing assumptions that are implicitly imposed by the problem solver, making it challenging to solve the problem analytically. For example, in the 9-dot problem, despite the absence of imposed assumptions, participants usually assume that the lines should be drawn within the square box. To solve the problem, the line must be drawn outside the square box, and participants might arrive at this conclusion through insight, analysis or sheer luck. While related studies primarily examined the occurrence of insight in such problems, this study focused on identifying the factors, especially learning characteristics, that facilitated the removal of implicit assumptions, rather than the reasons (such as insight, analysis, and sheer luck) that led to these assumptions being directly relaxed.

To accomplish this, a reinforcement learning (RL) framework was used in this study to provide a simple and rigorous account of problem solving and learning activities. The RL framework is supported by considerable empirical evidence, including neural signals in various cortical and subcortical structures that behave as predicted (Schultz et al., 1997; Glimcher and Rustichini, 2004; Hikosaka et al., 2006; Rangel et al., 2008). While this framework has been applied to studies of decision-making and learning in various social contexts (Delgado et al., 2005; Montague et al., 2006; Behrens et al., 2008; Hampton et al., 2008; Coricelli and Nagel, 2009; Bhatt et al., 2010; Yoshida et al., 2010), only a few studies have applied this to creative thinking (Harada, 2020a,b, 2021, 2023).

In this study the effect of learning characteristics measured by the RL framework in removing implicit assumptions and developing appropriate solutions was empirically investigated. Using our RL framework, which incorporates the prospect utility function, the exploitation/exploration ratio and the risk-taking and loss-taking attitudes can be estimated. The novelty of this approach in this study is simply that it allows us to examine the effects of learning traits such as exploitation/exploration ratio and loss-taking attitudes on insight problem solving, which would be impossible to assess without the computational model used in this study. Attitudes towards risk-taking have been extensively studied in the relevant literature by evaluating them using questionnaires. However, this method is subject to the subjective assessments of the participants, which could distort the measurement of risk attitudes. In contrast, risk attitude was determined on this study by estimating the underlying utility function based on objective behavioral data. Relevant literature emphasizes the role of risk-taking in fostering creativity as creative people are more likely to be motivated by challenging and risky situations (Albert, 1990; Perkins, 1990), suggesting a strong connection between risk-taking and creativity. Several empirical studies that examined this relationship reported that creativity and risk-taking are positively correlated (Eisenman, 1987; El-Murad and West, 2003; Dewett, 2007; Simmons and Ren, 2009; Tyagi et al., 2017 Harada, 2020a). However, Shen et al. (2018) found that, while low risk-taking was associated with convergent thinking, it was not significantly correlated with divergent thinking. Nevertheless, risk-taking and loss-taking in insight problems facilitate navigation through risky and unpromising sequences, which could help to relax or eliminate existing constraints that hinder problem solving and creative thinking. While related studies have investigated the effects of risk-taking on creativity, to our knowledge, the effects of loss-taking attitudes have not been examined because they could not be assessed without explicit consideration of a prospect utility function. This study tested the hypotheses that risk-taking and loss-taking are positively associated with performance in insight problem solving.

In addition, this study assigned two insight tasks (8-coin and 9-dot problems) and examined the possibility of knowledge transfer in insight problem solving, i.e., the contribution of experience in one task to problem solving in another task. The computational approach used in this study enabled the systematic assessment of the relative importance of the learning characteristics, especially risk- and loss-taking, for knowledge transfer in insight problem solving, which is not possible in the categorical sequential models that use multiple-comparison procedures for insight problem solving.

## Methods

### Participants

The insight tasks (8-coin and 9-dot problems) were assigned to 364 healthy undergraduates at Kobe University. Seven students were excluded because they did not participate in all tests, while the data of 32 students were dropped from the sample because they had previously experienced at least one of the two tasks. The remaining sample of 325 students was analyzed (111 women, age range = 18–26 years, SD = 0.47). The Ethics Committee of the Graduate School of Business Administration, Kobe University approved all experimental protocols in this study. The study conducted in compliance with the relevant guidelines and regulations. An informed consent form was signed by all participants and their parents (for those under 20 years of age).

### Experiments

In Test 1, participants completed a two-armed bandit (TAB) problem. In Test 2, they completed two insight tasks, 8-coin and 9-dot problems, in a randomly chosen order. For Tests 1 and 2, a TAB and two insight tasks were uploaded to our experimental server during class time. Each participant received Tests 1 and 2 at random. Programs for Tests 1 and 2 were developed, and the participants accessed the programs on the server from their PCs.

**Figure 1:**
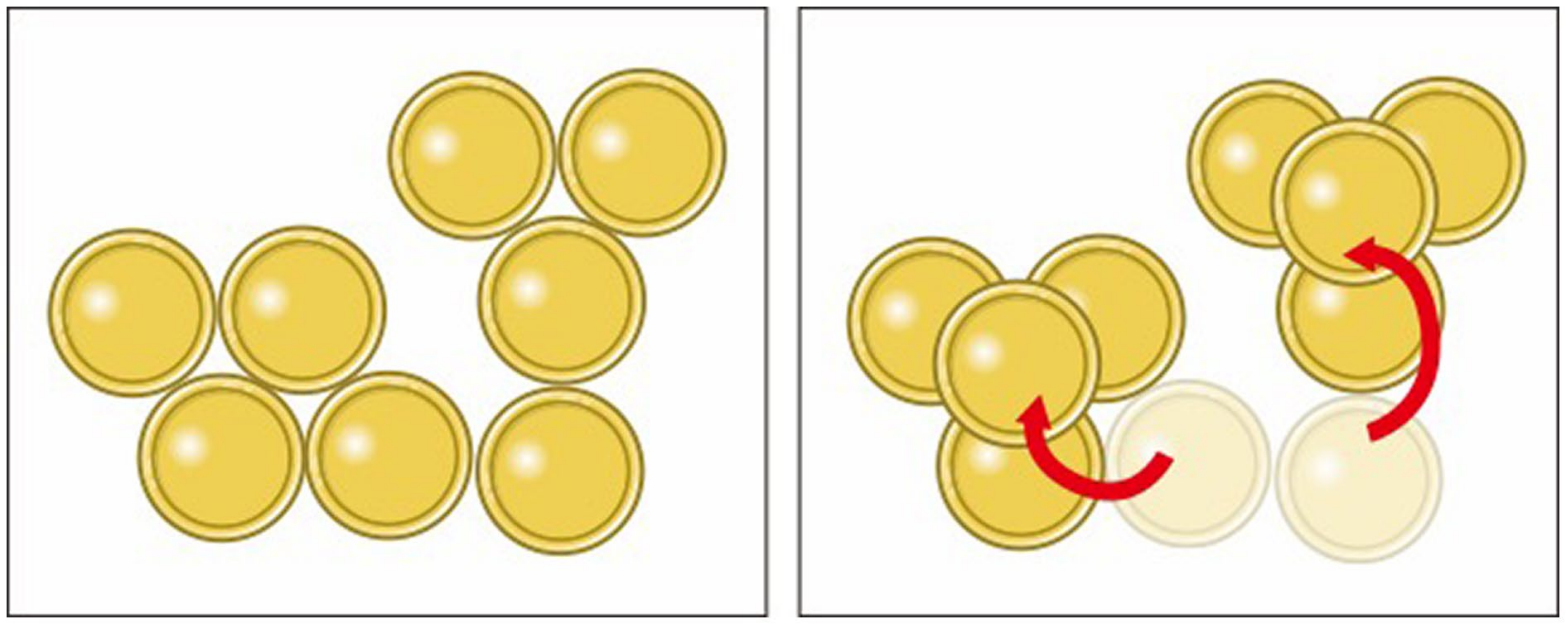
In the 8-coin problem, the figure on the left shows the participants. They were asked to move only two coins, so that each coin touched exactly three others. The figure on the right shows this solution.

In the 8-coin problem, the goal is to move only two coins from their respective starting positions such that each coin touches three other coins. Figure 1 shows the initial problem configuration and the final solution. To solve the problem requires switching from moving coins in two-dimensions to three-dimensions. Ormerod et al. (2002) reported low success rates without any hints.

**Figure 2:**
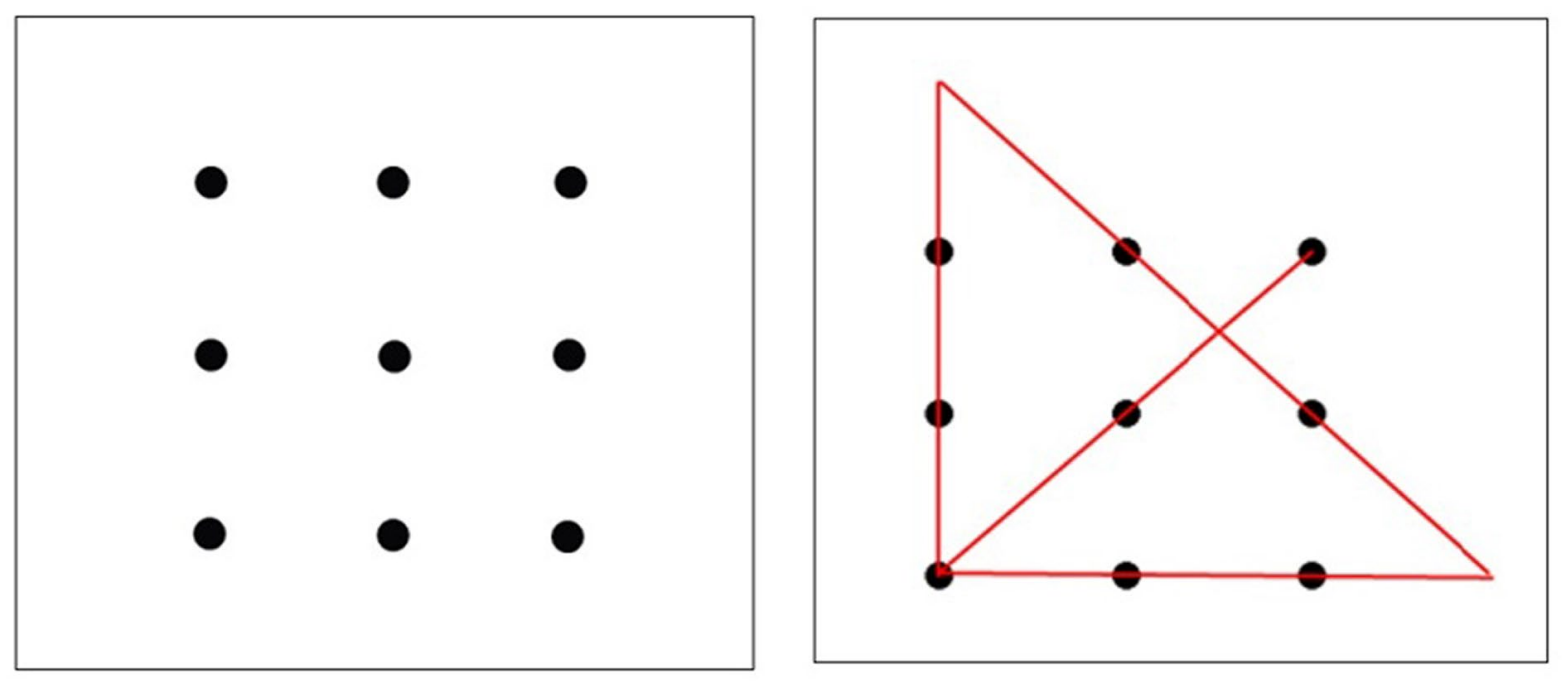
In the 9-dot problem, the figure on the left shows the participants. They were asked to connect all nine dots to four straight lines without lifting their pencils or retracing any lines. The figure on the right shows this solution.

In the 9-dot problem, the participant must connect all nine dots with four straight lines without lifting the pencil or retracing any lines. Figure 2 shows the initial problem configuration and the final solution. The insight required for this problem was to draw lines outside the 9-dot square box. The key to solving this problem lies in “thinking outside the box.” According to Weisberg and Alba (1981), all participants in their study reached an impasse, and none of them solved the problem. Even providing hints did not improve the situation. When they provided relatively detailed information on how to reach the solution, the success rate increased by the practice of solving simpler connect-the-dot problems. From this, they concluded that problem-specific experience was crucial to solving the problem.

Both the 8-coin and 9-dot problems had a 30-min time limit. If the participants were unable to solve the task within the time limit, the solution was displayed for 10 s and the program automatically proceeded to the next task (if it was the first problem) or Test 2 was terminated (if it was the second problem). If participants submitted a wrong answer, the message “incorrect” appeared immediately on the screen, followed by the solution.

The solutions to both problems require constraint relaxation. The 9-dot problem requires drawing lines outside the 9-dot square, whereas the 8-coin problem requires switching from two-dimensional to three-dimensional movement. Additionally, these are spatial puzzles (see Table 1), which are often categorized as spatial insight problems (Dow and Mayer, 2004). Therefore, learning transfer across two tasks was expected.

In our study, the success rates for the 8-coin and 9-dot problems were 31 and 70%, respectively (Table 2) which are significantly higher than those reported in similar studies. This difference could be attributed to the time limits for each problem. For example, the time limit for the 8-coin problem in Ormerod et al. (2002) was 6 min, whereas the time limits in this study were 30 min for both 8-coin and 9-dot problems. However, the successful participants completed the 8-coin problem in 1 min and 19 s and 9-point problem in 1 min and 44 s on average. Thus, the time limit in this study did not directly affect the success rates. A possible reason could be the occurrence of learning transfer. Although participants failed in the first test, they were able to succeed in the next test because they quickly learned that relaxing implicit assumptions is the key to success in the problems.

**Table 2:** Descriptive statistics.

	Mean	SD	1	2	3	4	5	6	7	8	9
**1. Success in 8-coin**	0.31	0.46	—								
**2. Success in 9-dot**	0.7	0.46	0.18***	—							
**3. β (Inverse temperature)**	0.85	1.06	−0.03	−0.01	—						
**4. μ (risk aversion in gains)**	0.48	0.28	−0.05	−0.09	0.04	—					
**5. ν (risk-seeking in losses)**	0.52	0.3	−0.01	0.06	−0.05	−0.05	—				
**6. α + (learning late)**	0.42	0.27	0.05	0.02	−0.24**	−0.02	0.01	—			
**7. α- (learning late)**	0.59	0.29	−0.14**	0.05	0.08	0.08	0.04	0.02	—		
**8. λ (loss aversion)**	0.51	0.28	−0.03	−0.12**	−0.02	0.04	−0.06	−0.04	−0.04	—	
**9. Φ (autocorrelation control)**	−12.57	213.86	0.04	0.10	0.04	−0.09	0.06	−0.03	0.02	0.06	
**10. TAB performance**	−0.7	10.67	−0.02	−0.07	0.08	−0.04	−0.01	0.06	−0.08	−0.08	−0.05

*N* = 325. **p < 0.05, ***p < 0.01.

### Q-learning model

**Figure 3:**
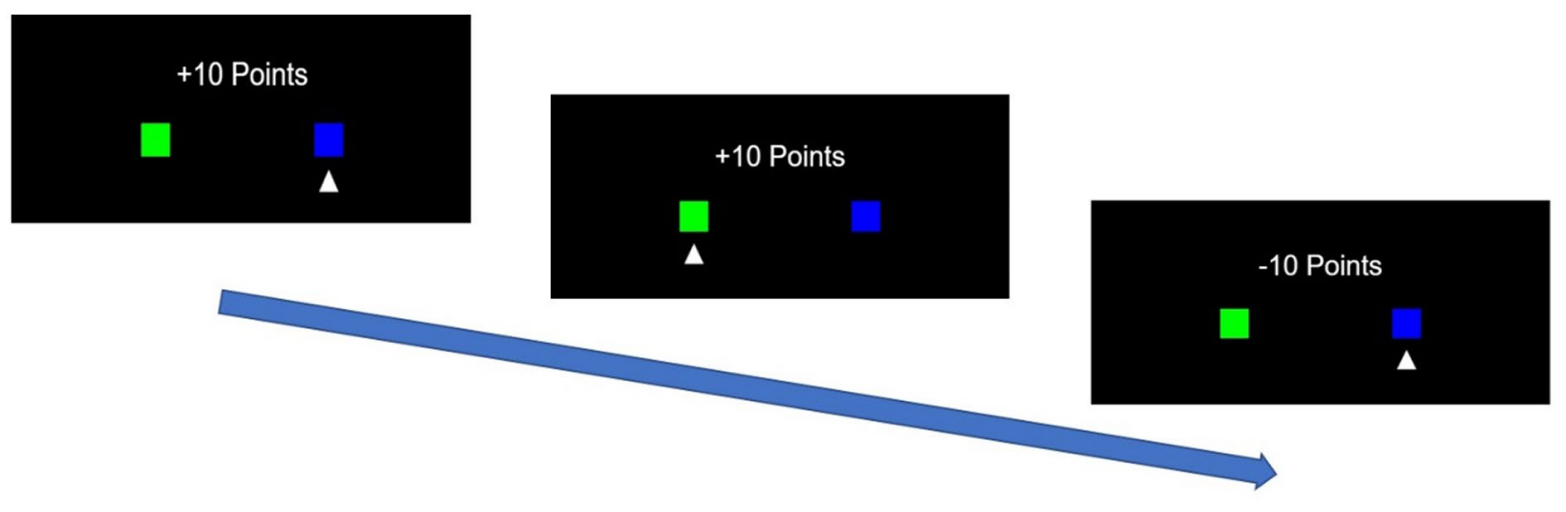
Example of a trial in the two-armed bandits (TAB) in which the participant chose the right box first, then the left, and finally the right box, with rewards of 10, 10, and −10 points, respectively.

To account for decision-making in the TAB, a simple Q-learning reinforcement learning algorithm was used in this study (Watkins and Dayan, 1992). In Test 1, participants selected either the right or left box on the screen (Figure 3). Upon selection, participants were immediately awarded either 10 or − 10 points. The goal of this test was to maximize the sum of the rewards over a series of 100 choices. The probability of gaining 10 points was higher for one box (70%) and lower for the other (30%). These probabilities were switched twice over 100 choices to eliminate the possibility of learning convergence, where the participants learn the box with the higher probability of gaining 10 points and choose that box in the future. For the first 30 choices, the right and left boxes had a 70 and 30% probability of gaining 10 points, respectively. From the 31st to the 70th choice, the probabilities switched such that the probability of earning 10 points for the right and left boxes fell to 30 and 70%, respectively. Subsequently, for the last 30 choices, the probabilities of the right and left boxes returned to initial levels of 70 and 30%, respectively. These shifts in probabilities were built in to prevent participants from continuing to select the same deck with a higher expected reward in the early stages of the 100 trials.

Q-learning assumes that a decision-maker calculates the action value for choice *i* at trial *t* (*i =* right or left box), which is denoted by $Q_{i}\left(t\right)$ as


(1) $$Q_{i}\left(t+1\right)=\{\;\begin{array}{c} Q_{i}\left(t\right)+\alpha^{+}\delta \left(t\right)+\phi \;\mathrm{if}\;\delta \left(t\right)\geq 0,\\ \;Q_{i}\left(t\right)+\alpha^{-}\delta \left(t\right)+\phi \;\mathrm{if}\;\delta \left(t\right)<0\text{,} \end{array}$$

with


(2) $$\delta \left(t\right)=U\left(R_{i}\left(t\right)\right)-Q_{i}\left(t\right)\text{,}$$

(3) $$U\left(R_{i}\left(t\right)\right)=\{\begin{array}{c} R_{i}{\left(t\right)}^{\mu }\;\;\mathrm{if}\;R_{i}\left(t\right)>0,\\ -\lambda {\left(-R_{i}\left(t\right)\right)}^{\nu }\;\;\mathrm{if}\;R_{i}\left(t\right)<0\text{,} \end{array}$$

where $R_{i}\left(t\right)$ is the reward associated with choice i at trial t , either 10 or − 10 points, and $\delta \left(t\right)$ is the reward prediction error. $\alpha^{\pm }$ indicates the learning rate, which measures the sensitivity to gains and losses when updating the action value. ϕ is added to Equation 1, because participants may tend to make the same choice over time. This autocorrelation of choices could bias the magnitude of the learning rate $\alpha^{\pm }$ (Katahira, 2018). ϕ was added to correct this bias.

Following Harada (2023), the prospect utility function (Tversky and Kahneman, 1986) was incorporated $\mathrm{in}\;U\left(R_{i}\left(t\right)\right)$ because it facilitates the measurement of risk and loss attitudes without additional paper and pencil tests. μ and ν in Equation 3 measure the degree of risk aversion and risk-taking, respectively. In this specification, risk-taking (aversion) is associated with lower (higher) μ and higher (lower) ν . λ evaluates losses relative to gains, which is usually referred to as loss aversion. A higher λ implies that agents want to avoid losses. Note that λ measures sensitivity to negative rewards, whereas risk attitudes evaluate sensitivity to changes in rewards.

When box j is not chosen by the decision-maker, its action value remains the same, such that


(4) $$Q_{j}\left(t+1\right)=Q_{j}\left(t\right)$$

Faced with the action values of the two boxes, it is assumed that the decision maker chooses one of the two according to the SoftMax decision rule.


(5) $$P\left(a\left(t\right)=i\right)=\frac{\exp\left(\beta Q_{i}\left(t\right)\right)}{\sum_{j=1}^{2}\exp\left(\beta Q_{j}\left(t\right)\right)}\text{,}$$

where $a\left(t\right)$ represents the choice made at trial t and $P\left(a\left(t\right)=i\right)$ refers to the probability of choosing box i at trial t . Parameter β is the inverse temperature indicating the relative strength of exploitation versus exploration (exploitation/exploration ratio), which was originally proposed in the RL framework (Sutton and Barto, 2018). Exploitation refers to the optimization of current tasks under existing information and memory conditions, whereas exploration implies wider, and sometimes random, searches and trials. Consequently, exploitation and exploration usually generate different solutions, resulting in a trade-off between the two. A higher inverse temperature indicates that the decision-maker chooses the box with the higher *Q* values. In contrast, a lower inverse temperature suggests that the choice is more likely to be made randomly, independent of the *Q* values.

In this study, it was hypothesized that this Q-learning model could also specify creative thinking processes in insight tasks. In the 9-dot problem, Weisberg and Alba (1981) highlighted the importance of providing relatively detailed information about the problem to improve the success rate. In particular, problem-specific knowledge is required to solve a problem. In the 8-coin problem, Ormerod et al. (2002) emphasized the importance of current constraints and preferred strategic moves when changing the search direction. These findings suggest that existing beliefs and knowledge regarding strategic activities and directions play a role in finding solutions, which can be formalized as the action values of each option. The action values are derived from an individual’s prior beliefs and experiences, as specified in Equations 1–4.

Moreover, unrealized options can be represented by options that have the maximum possible action values after the “a-ha” moment and zero action values prior to it. The above Q-learning model may seem to represent only incremental learning while the “a-ha” moment entails sudden learning wherein a zero-valued option swiftly increases to its maximum value. However, this sudden shift in the option values could be triggered by a lower value of the inverse temperature β (exploration) in Equation 5. A random choice of a low valued option might result in an extremely higher reward $R_{i}\left(t\right)$ and $\delta \left(t\right)$ in Equation 2, leading to an immediate shift in its *Q* value in Equation 1. Thus, the Q-learning model described above could be applied not only to the TAB, but also to the 8-coin and 9-dot problems. The research strategy in this study was to estimate the parameter values of the Q-learning model from the TAB in Test 1 and evaluate their effects on the performance of the two insight tasks in Test 2.

### Estimation method

The parameters specified in Equations 1–5 were estimated by optimizing the maximum *a posteriori* objective function.


$$\hat{\theta }=\mathrm{argmax}\;p\left(D_{s}|\theta_{s}\right)p\left(\theta_{s}\right)\text{,}$$

where $p\left(D_{s}|\theta_{s}\right)$ is the likelihood of data $D_{s}$ for subject s under the condition of the parameters $\theta_{s}=\left\{\beta^{S},\mu^{S},\nu^{S},{\alpha^{\pm }}^{S},\lambda^{S},\phi^{S}\right\}$ . $p\left(\theta_{s}\right)$ is the prior probability of $\theta_{s}$ . Note that $\alpha^{\pm }$ should be bound between 0 and 1 and β,μ,ν,andλ, take non-negative values. Following a standard procedure in Bayesian statistics, the priors for $\alpha^{\pm }$ were specified as beta distributions with shape parameters of 2 and 2, and the priors for β,μ,ν,andλ were gamma distributions, f, with a shape parameter of 2 and a scale parameter of 3. ϕ was assumed to follow a standard normal distribution with a mean of 0 and variance of 1.

## Results

This section examines the effects of the learning characteristics in the Q-learning model. The descriptive statistics (mean, SD, and correlation) for all the variables used in the empirical analyses are listed in Table 2.

For this purpose, the parameters of inverse temperature (β), the risk-aversion index for gains (μ), the risk-taking index for losses (ν), learning rates ( $\alpha^{\pm }$ ), loss aversion ( λ ), and autocorrelation ( ϕ ) were estimated from the data obtained in the TAB by the MAP estimation described above using R and the Rsolnp and tidyverse libraries. Regression analyses were then performed on the determinants of success in the 8-coin and 9-dot problems. Performance in TAB (TAB performance) was also added as a regressor. As the measures indicating success in these two tasks were dummy variables (1 and 0 for success and failure, respectively), the probit regression method was used to maintain statistical consistency. The results are listed in Table 3.

**Table 3:** Probit regression results (SE in parentheses).

	**Success**			
**Variables**	**8-coin problem**		**9-dot problem**	
	**(1)**	**(2)**	**(3)**	**(4)**
**Constant terms**	−0.07	−0.53	0.78*	0.53
	(0.31)	(0.35)	(0.32)	(0.33)
**Success in 8-coin**				0.59***
				(0.18)
**Success in 9-dot**		0.59***		
		(0.18)		
**β (inverse temperature)**	0.00	0.00	0.00	0.00
	(0.07)	(0.07)	(0.07)	(0.07)
**μ (risk aversion in gains)**	−0.15	−0.08	−0.37	−0.34
	(0.27)	(0.27)	(0.27)	(0.27)
**ν (risk-seeking in losses)**	−0.05	−0.09	0.21	0.23
	(0.25)	(0.25)	(0.25)	(0.26)
**α + (learning rate)**	0.28	0.26	0.16	0.11
	(0.28)	(0.28)	(0.28)	(0.29)
**α- (learning rate)**	−0.63**	−0.69***	0.16	0.31
	(0.25)	(0.26)	(0.26)	(0.27)
**λ (loss aversion)**	−0.18	−0.07	−0.62**	−0.63**
	(0.27)	(0.27)	(0.27)	(0.27)
**Φ (autocorrelation)**	0.00	0.00	0.01	0.01
	(0.01)	(0.01)	(0.00)	(0.00)
**TAB performance**	0.00	0.00	−0.01	−0.01
	(0.01)	(0.01)	(0.01)	(0.01)
**AIC**	409.34	400.02	396.15	386.71

*N* = 325. *p < 0.10, **p < 0.05, ***p < 0.01.

Columns (1) and (2) of Table 3 show the results for the 8-coin problem. Column (2) contains a dummy variable indicating success in the 9-dot problem. In both columns, the learning rate for the negative reward prediction errors ( $\alpha^{-})$ exerted a negative effect. This suggests that successful individuals tend to respond to negative results positively in updating the Q value. Moreover, column (2) clearly indicates that successful individuals in the 9-dot problem were more likely to be successful in the 8-coin problem. A possibility of learning transfer exists between these two insight tasks, as they belong to the same category of insight problems, so-called the spatial insight problems (Dow and Mayer, 2004). The ability to solve the 9-dot problem was carried over to the 8-coin problem, suggesting that problem-solving ability is not limited to problem-specific knowledge.

Columns (3) and (4) show the results for the 9-dot problem. Column (4) contains a dummy variable indicating success in the 8-coin problem. In both columns, loss aversion λ exerted a negative effect, implying that successful individuals in the 8-coin problem tend to react positively to negative rewards. Furthermore, 8-coin problem success had a positive effect on 9-dot-problem success. Thus, problem solving ability for the 8-coin problem also contributed to the 9-dot problem.

These results imply that the determinants of success in the two insight tasks differ completely in terms of the learning characteristics of the Q-learning model. Nevertheless, the negative effects of $\alpha^{-}$ and λ suggest that insight problem solving must respond positively in updating the Q value. Moreover, the results indicated that problem solving abilities in both tasks were closely related.

However, these results do not account for the order effect of the two insight tasks. If something is learned from an insight task, the lessons could provide useful guidance in the next insight task. The sample was split into two subsamples to comprehend this order effect. In these subsamples, participants performed one of the two tasks for the second time such that they had already experienced another insight task. The results are listed in Table 4.

**Table 4:** Probit regression results for the second-time tasks (SE in parentheses).

	**Success**			
**Variables**	**8-coin problem**		**9-dot problem**	
	**(1)**	**(2)**	**(3)**	**(4)**
**Constant terms**	0.60	0.16	0.33	0.11
	(0.45)	(0.52)	(0.44)	(0.45)
**Success in 8-coin**				0.74***
				(0.27)
**Success in 9-dot**		0.48*		
		(0.27)		
**β (inverse temperature)**	−0.02	−0.01	0.13	0.14
	(0.10)	(0.11)	(0.12)	(0.12)
**μ (risk aversion in gains)**	−0.63	−0.59	−0.29	−0.33
	(0.38)	(0.38)	(0.38)	(0.38)
**ν (risk-seeking in losses)**	−0.15	−0.20	0.00	0.00
	(0.36)	(0.36)	(0.35)	(0.37)
**α + (learning rate)**	0.23	0.24	0.38	0.37
	(0.40)	(0.41)	(0.40)	(0.41)
**α- (learning rate)**	−0.50	−0.45	0.62*	0.85**
	(0.37)	(0.37)	(0.36)	(0.38)
**λ (loss aversion)**	−0.78**	−0.73	−0.80**	−0.91**
	(0.38)	(0.38)	(0.38)	(0.39)
**Φ (autocorrelation)**	0.01	0.01	0.00	0.00
	(0.01)	(0.01)	(0.00)	(0.00)
**TAB performance**	−0.01	−0.01	−0.01	−0.01
	(0.01)	(0.01)	(0.01)	(0.01)
**AIC**	219.34	217.91	214.3	208.15

*N* = 325. *p < 0.10, **p < 0.05, ***p < 0.01.

First, based on the results in Table 4, success in the previous insight task positively affected success in the following insight task. Second, a positive effect of $\alpha^{-}$ is observed in the 9-dot problem, but it is no longer significant in the 8-coin problem. Interestingly, in contrast to the previous results, all columns in Table 4 show significant negative effects of loss aversion λ . In both the 8-coin and 9-dot problems, the success of the insight tasks in the second time critically depended on their insensitivity to avoid reward losses. In particular, successful individuals were more willing to accept losses than to avoid them. Lower loss aversion appears to be critical for transferring what has been learned to other tasks.

To check the robustness of this result, subsamples in which participants undertook insight tasks for the first time were also examined. In this analysis, no common effects of learning characteristics were observed between the two tasks. The significant parameters were $\alpha^{-}$ for the 8-coin problem and a constant term for the 9-dot problem. The success rates for the first and second-time tasks were 0.25 and 0.36 for the 8-coin problem ( $\chi^{2}=$ 4.84, *p* = 0.03) and 0.64 and 0.77 for the 9-dot problem ( $\chi^{2}=$ 5.74, *p* = 0.02), respectively, indicating that prior learning was transferred to the next task. Hence, for this learning transfer to occur, loss-taking, rather than loss aversion, played a critical role in both insight tasks.

## Discussion

In this study, a novel methodology for studying insight problem solving was proposed and the effects of learning parameters specified in the Q-learning model in insight problem solving performance were investigated. To the best of our knowledge, this is one of the first attempts to use a computational approach to study insight tasks, such as the 8-coin and 9-dot problems. Although there are several studies that have empirically investigated insight problem solving and cognitive strategies, most of them have not explicitly modeled the underlying mechanism of problem solving in insight tasks. In addition to the categorical conceptual models of insight thinking processes (Ohlsson, 1992; Finke et al., 1999; Knöblich et al., 1999; Perkins, 2000; Weisberg, 2015), a frequently used method in related studies was retrospective reporting such as feeling-of-warmth rating, in which participants were asked to assess “how warm/close do you feel you are to the solution?” or respond to a verbal protocol, in which they were asked what they are thinking while working on the solution (Chu and Macgregor, 2011). Evidently, these methods are subjective and unreliable. In addition, retrospective reporting during the tasks themselves has been reported to affect performance (Berardi-Coletta et al., 1995), which could bias the results, and make it more difficult to assess the effect of the underlying mechanism. Undoubtedly, pertinent research has also investigated the preconditions for insight such as mind-wandering thoughts (Zedelius and Schooler, 2015; Gable et al., 2019) and looking away behavior (Salvi and Bowden, 2016) after being unsuccessful at solving a problem. However, these preconditions have not been integrated into a coherent model of insight problem solving.

In contrast, a computational approach to insight problem solving was described in this study. This algorithm allows for a more accurate understanding of the processes that occur while people solve insight problems, as it can identify the parameters that influence learning. In particular, detailed individual differences in learning traits could be examined in insight problem solving using this approach which could further our understanding on insight problem solving processes and help enhance creative thinking. Of course, it must be noted that our computational approach was not directly applied to insight problem solving. Instead, the learning parameters were estimated in the TAB tasks. Nevertheless, we believe that the Q-learning framework could also be applied to insight problem solving by interpreting insight as a sudden shift of a low- or zero-valued option triggered by exploration.

It should also be noted that the proposed Q-learning model replicates actual brain activity as it is based on the underlying neural mechanism. This RL framework is supported by a growing number of studies on neural mechanisms (Schultz et al., 1997; Glimcher and Rustichini, 2004; Hikosaka et al., 2006; Rangel et al., 2008). For example, research supports the existence of a connection between behavior and dopamine neurons in the midbrain of humans and monkeys that encode reward-prediction errors (Schultz et al., 1997; Bayer and Glimcher, 2005; Cohen et al., 2012). The Q-learning model proposed in this study belongs to this class of models that can be used to model brain activity. Thus, the Q-learning model suffers less from the arbitrariness and *ad hoc* nature typically observed in the related conceptual models.

Regarding the hypotheses that risk-taking and loss-taking improve performance in insight problems, no significant effects of risk-taking were observed. This result supports the findings of Shen et al. (2018), according to which risk-taking was not significantly correlated with divergent thinking. In contrast, loss-taking was positively related to performance in the 9-dot problem but not in the 8-coin problem. These results suggest that loss-taking, rather than risk-taking, was partially responsible for insight problem solving performance.

However, when the learning transferability between the two problems is taken into account, loss-taking assumes a substantial role in both tests. The performance in the second insight problem solving improved with loss-taking attitudes. Therefore, the hypothesis must be modified to the effect that loss-taking is positively related to performance in insight problems under learning transfer.

The learning transfer has also been confirmed in related studies. Ansburg and Dominowski (2000) found that insight problem solving can be construed as a general strategic thinking skill for which training is useful. Chrysikou (2006) also reported that additional general training that does not directly target insight problems can improve insight problem solving. However, several studies questioned the generalizability of problem-solving ability. They claimed that training for one insight problem is not transferable to other insight problems (Dow and Mayer, 2004; Cunningham and Mac Gregor, 2008). One possible reason for these differences could be that different types of insight problems require different cognitive abilities (Chu and Macgregor, 2011). In this study, the learning transfer could have occurred between the 8-coin and 9-dot problems because of their similarity. In the debate on the transferability or learning in insight problems, this research made a unique contribution by identifying the factor that facilitates learning transfer, namely, the attitude toward loss-taking. In addition to the differences in the nature of insight problems, a lack of this attitude may prevent learning transfer. Hence, individual differences in learning characteristics play a role in establishing learning transfer across insight problems.

The literature on analogical transfer in insight problems argues that providing a problem analogy, such as similes, metaphors, and case-based reasoning, improves solution rates (Reeves and Weisberg, 1994). A positive attitude towards failure (loss-taking in the context of Q-learning) could facilitate this analogical transfer. If lessons from failure are appropriately generalized in analogies or case-based reasoning, it could serve as a guide. Accepting and learning from failure leads to the creation of useful analogies that reflects previous experiences of failure to overcome the next insight problem.

According to prospect theory, people are willing to take risks to avoid losses (Tversky and Kahneman, 1992). One of the implications of this study is that the creativity of those who do not attempt to avoid losses can be enhanced. Although loss-taking only partially responsible for performance in insight problems, it facilitated problem solving in both 8-coin and 9-dot problems under learning transfer. It is our conviction that this attitude can often be trained such that loss-averting individuals strive for more loss-seeking. Even if this is difficult, appropriate incentives can be created to encourage loss-seeking by rewarding (constructive) failure. For example, a global mobility company, Honda, introduced a challenging goal system in which employees were evaluated on the basis of processes rather than performance (results). The criteria for process evaluation included the number of instances in which employees experienced constructive failure (Harada, 2010). Alternatively, reducing actual losses due to failure by introducing simulations, virtual experiments or rapid prototyping could also improve creativity (Ries, 2011).

However, the results of this study have several limitations. First, the Q-learning model was only applied to TAB tasks and the effects of its learning parameters over different insight problems were assessed. Therefore, while we argued that the Q-learning model could model the insight problem solving activity, the computational approach in this study was limited in the sense that it was not applied for analyzing insight problem solving directly. When the cognitive activities in the TAB and insight problem share the same mechanism, the results showed the direct effect of learning traits in insight problem solving. However, even if non-insight and insight problem solving follow the Q-learning mechanism, it is possible that the parameter values differ in the different problems (even across different insight problems). It is evident that this possibility should be further investigated in future studies by applying the computational approach directly to insight problems. To achieve this, more sophisticated computer programs must be developed to track detailed thought processes during insight problem solving.

Second, only two insight problems were investigated in this study. However, it would be more interesting to examine the learning transfer not only across similar types of insight problems, but also for different types of insight problems. A more systematic study on a variety of insight problems will reveal the domain-free determinants of learning transfer in insight problems.

Third, this study examined the determinant of performance in insight problem solving, In related studies, the occurrence of insight has typically been investigated using retrospective reports after insight solving (Chu and Macgregor, 2011). However, as described above, this method is subjective and unreliable. As a result, this study did not examine whether insight actually occurred for each participant. Therefore, the results of this study might also reflect solutions without insight. Hence, the results should be interpreted as a determinant of performance of so-called “insight problems” in which no distinction was made as to whether insight actually occurs or not. In future studies, we should more objectively determine whether insight occurs or not to examine the determinant of problem solving with insight, which would probably require a neuroscientific approach.

Finally, we point out that our results critically depend on the cultural and social background of the participants. Results may differ when similar experiments are conducted in different contexts, although any psychological study is subject to this type of limitation. Even if different results are obtained, we believe that the computational approach to insight problem solving and the simple Q-learning framework in this study remain valid and useful.

## Conclusion

This study examined the effects of learning traits on insight problem solving, using a computational approach to uncover the correlational factors linked with insight problem solving. The result revealed that positively reacting to loss and errors is a crucial characteristic for successful insight problem solving in both 8-coin and 9-dot problems, facilitating analogical transfer between the two tasks and improving performance. This assessment was made possible by implementing a simple Q-learning model and estimating learning parameters. To the best of our knowledge, this study is one of the few attempts to apply the RL framework to insight problem solving and learning transfer.

## Data availability statement

The raw data supporting the conclusions of this article will be made available by the authors, without undue reservation.

## Ethics statement

The studies involving humans were approved by The Ethics Committee of the Graduate School of Business Administration, Kobe University. The studies were conducted in accordance with the local legislation and institutional requirements. The participants provided their written informed consent to participate in this study.

## Author contributions

TH: Conceptualization, Data curation, Formal analysis, Funding acquisition, Investigation, Methodology, Project administration, Resources, Software, Supervision, Validation, Visualization, Writing – original draft, Writing – review & editing.
